# The acute effects of exercise intensity and inorganic nitrate supplementation on the Buckberg ratio in postmenopausal females

**DOI:** 10.1113/EP093920

**Published:** 2026-07-07

**Authors:** Cassandra C. Derella, Austin C. Hogwood, Joaquin Ortiz de Zevallos, Nathan R. Weeldreyer, Kaitlin M. Love, Arthur Weltman, Jason D. Allen

**Affiliations:** ^1^ Department of Kinesiology University of Virginia Charlottesville Virginia USA; ^2^ Department of Cardiovascular Medicine Robert M. Berne Cardiovascular Research Center Charlottesville Virginia USA; ^3^ Department of Molecular Medicine and Surgery Karolinska Institute Stockholm Sweden; ^4^ Faculty of Nursing, College of Health Sciences University of Alberta Edmonton Alberta Canada; ^5^ Division of Endocrinology and Metabolism, School of Medicine University of Virginia Charlottesville Virginia USA; ^6^ Advent Health Translational Research Institute Orlando Florida USA; ^7^ Department of Medicine University of Virginia Charlottesville Virginia USA

**Keywords:** aortic haemodynamics, female cardiovascular health, nitric oxide bioavailability

## Abstract

Menopause increases coronary heart disease risk, in part through reduced oestrogen‐mediated nitric oxide (NO) bioavailability. The Buckberg ratio, an index of myocardial oxygen supply–demand balance, in which higher values reflect more favourable myocardial perfusion, is lower in postmenopausal females (PMFs) than in age‐matched males. High‐intensity exercise enhances NO production and endothelial function more than moderate‐intensity exercise, yet PMFs often exhibit blunted vascular adaptations. Inorganic nitrate (NO_3_^−^) supplementation increases NO bioavailability and improves endothelial function, but its effect on the Buckberg ratio is unknown. We hypothesised that the Buckberg ratio would decline after exercise in an intensity‐dependent manner and that this reduction would be attenuated by NO_3_^−^ supplementation. In a double‐blind, randomized design, 24 PMFs (60 ± 5 years; 27.5 ± 5.9 kg/m^2^; 10 ± 5 years postmenopausal; peak oxygen consumption = 23.9 ± 4.5 mL/kg/min) consumed nitrate‐rich beetroot juice (BRJ; *n* = 12) or nitrate‐depleted placebo (PL; *n* = 12) for ∼2 days, with a final dose 2 h before testing. The Buckberg ratio was measured at rest and every 30 min for 3 h following calorically matched high‐intensity exercise (HIE), moderate‐intensity exercise (MIE) and non‐exercise control (CON) conditions using pulse wave analysis (SphygmoCor) and was calculated from the derived central aortic pressure waveform as the ratio of diastolic to systolic pressure–time integrals, reflecting myocardial oxygen supply relative to demand. Venous blood collected pre‐ and post‐supplementation assessed plasma NO_3_^−^ and nitrite (NO_2_^−^). BRJ increased plasma NO_3_^−^ (64.9 ± 20.8 vs. 1114.2 ± 176.2 µM, *P* < 0.001) and NO_2_^−^ (118.3 ± 57.9 vs. 356.8 ± 114.0 nM, *P* < 0.001). The Buckberg ratio was unchanged during CON but declined after MIE (*P* < 0.001) and HIE (*P* < 0.001). BRJ attenuated this decline following HIE (*P* < 0.001), but not MIE. These findings indicate that acute exercise reduces the Buckberg ratio in PMFs in an intensity‐dependent manner, and NO_3_^−^ supplementation might help to preserve coronary perfusion during higher‐intensity exercise stress.

## Abstract (highlights)

- **What is the central question of this study?**
  Does acute exercise intensity influence the Buckberg ratio (an index of myocardial perfusion) in postmenopausal females, and can inorganic nitrate supplementation attenuate any exercise‐induced reductions
- **What is the main finding and its importance?**
  Acute exercise reduced the Buckberg ratio in an intensity‐dependent manner, with the greatest decline following high‐intensity exercise. Inorganic nitrate supplementation attenuated this reduction, suggesting improved coronary perfusion during exercise stress and highlighting dietary nitrate as a potential strategy to mitigate myocardial perfusion imbalance in postmenopausal females.

## 1 INTRODUCTION

Cardiovascular disease remains the leading cause of death among females, and coronary heart disease risk rises substantially after menopause (El Khoudary et al., 2020). By 2030, >1.2 billion females worldwide are expected to be postmenopausal (Sussman et al., 2015). The transition into menopause is marked by a decline in circulating oestrogen, a hormone essential for vascular protection through its regulation of endothelial nitric oxide (NO) production, antioxidant defence and anti‐inflammatory signalling (Somani et al., 2019). As oestrogen levels fall, NO bioavailability decreases, leading to endothelial dysfunction, increased arterial stiffness, a potential reduction in myocardial perfusion and a heightened risk of coronary heart disease risk in postmenopausal females (PMFs) (Egelund et al., 2019; Raj et al., 2023).

The Buckberg subendocardial viability ratio (Buckberg ratio) is a non‐invasive estimate of myocardial perfusion (Chirinos, 2022; Tsiachris et al., 2012). The Buckberg ratio is derived from analysis of the arterial pulse wave and reflects coronary perfusion pressure during diastole relative to cardiac workload during systole (Chirinos, 2022). Lower Buckberg ratio values are associated with reduced coronary perfusion and are predictive of myocardial ischaemia and adverse cardiac events (Laurent et al., 2006; Tsiachris et al., 2012). Notably, PMFs exhibit a lower Buckberg ratio compared with age‐matched males, suggesting a sex‐specific vulnerability in myocardial oxygen delivery and perfusion efficiency after menopause (Laugesen et al., 2016; Tagawa et al., 2022). This vulnerability might be attributable, in part, to the loss of oestrogen. As such, interventions to improve the Buckberg ratio in PMFs might yield considerable clinical benefit.

Exercise is a well‐established intervention to promote vascular health and reduce coronary heart disease risk (Saz‐Lara et al., 2021), but its effects on myocardial perfusion in PMFs remain poorly understood. Acute exercise can transiently reduce the Buckberg ratio owing to increases in heart rate and systolic pressure, particularly at higher intensities (Doonan et al., 2011). Although generally well tolerated in healthy adults, this reduction might be more concerning in PMFs, who already exhibit impaired endothelial function (Guzic‐Salobir et al., 2001) and increased arterial stiffness (Waddell et al., 2001), heightening their vulnerability to myocardial supply–demand mismatches during stress. This raises important questions about how to maximize the cardiovascular benefits of exercise while minimizing potential risks in PMFs. One potential approach is to increase bioavailable NO prior to exercise.

Oral inorganic nitrate supplementation is a recognized approach to restore NO bioavailability, but its effects in PMFs are not well studied (Lidder & Webb, 2013). Unlike the enzymatic pathway for endothelium‐dependent NO production, the nitrate–nitrite–NO pathway bypasses this dysfunctional pathway in PMFs by using enterosalivary reduction of dietary inorganic nitrate to generate NO (Woessner et al., 2016). Beetroot juice (BRJ), a rich source of inorganic nitrate, has been shown to increase plasma nitrate (NO_3_^−^) and nitrite (NO_2_^−^) concentrations and improve vascular function in a variety of healthy and clinical populations (Chirinos & Zamani, 2016; Delgado Spicuzza et al., 2024; Volino‐Souza et al., 2022; Walker et al., 2019). In conditions of low oxygen tension or acidosis, such as during exercise, plasma nitrite can be reduced readily to bioactive NO through several one‐electron reduction pathways. This pathway might be particularly promising for PMFs to preserve vascular homeostasis and myocardial perfusion under stress.

In support of the above, recent work from our laboratory demonstrated that acute BRJ supplementation improves postexercise brachial artery endothelial function in PMFs, particularly when combined with high‐intensity exercise (HIE) (Hogwood et al., 2023). However, the effects of oral inorganic nitrate supplementation on coronary perfusion, as assessed by the Buckberg ratio, remain unexplored in this population.

The purpose of the present study was to determine whether the Buckberg ratio is reduced following calorie‐matched acute exercise in an intensity‐dependent manner in heathy PMFs. We also aimed to assess whether inorganic nitrate supplementation can attenuate these reductions. We hypothesised that the Buckberg ratio would decrease following exercise, particularly after high‐intensity exercise, and that BRJ supplementation would mitigate this reduction, supporting its potential to preserve coronary perfusion under stress in PMFs.

## 2 MATERIALS AND METHODS

### 2.1 Experimental design

**FIGURE 1:**
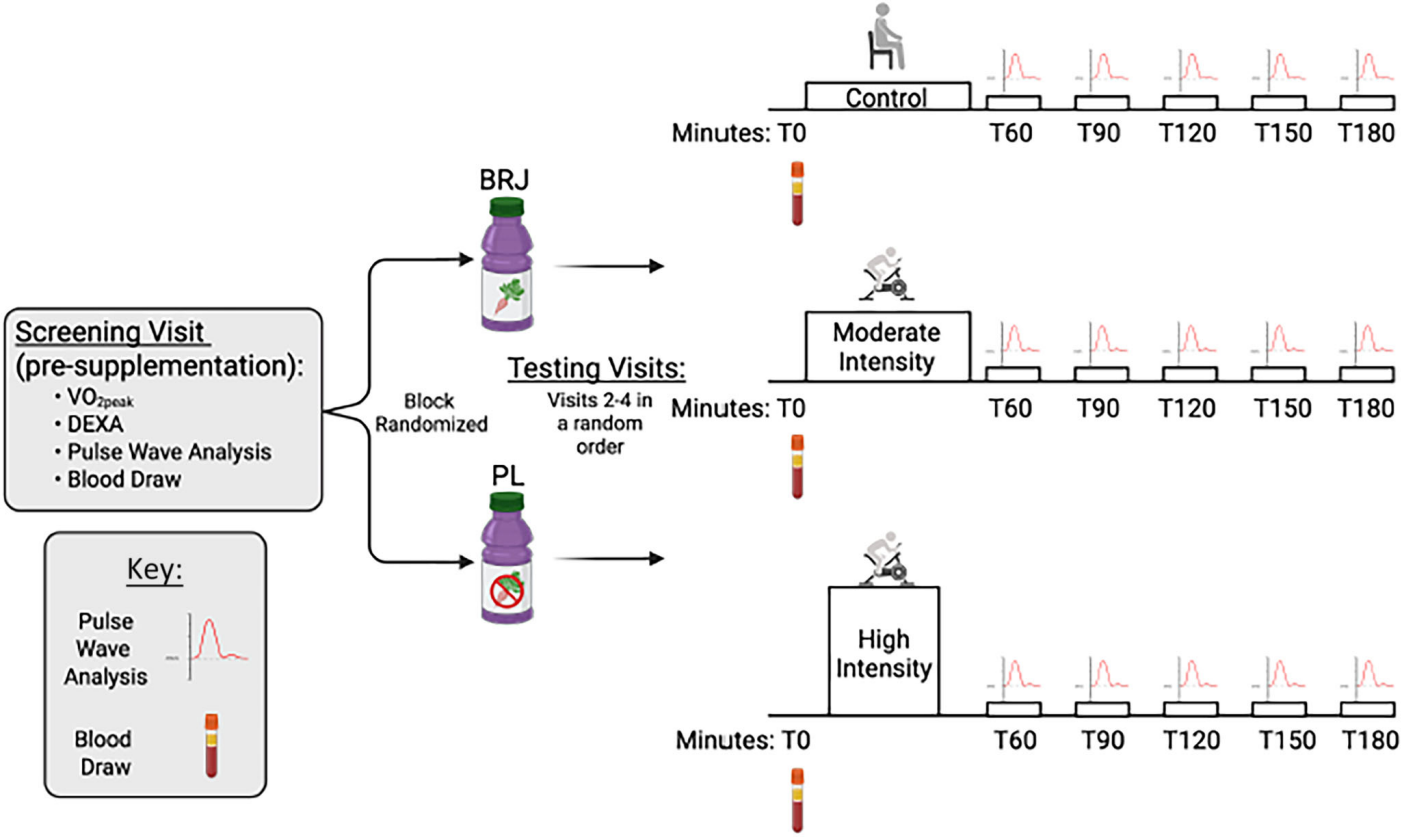
Schematic diagram of study protocol. BRJ, beetroot juice (*n* = 12); PL, placebo (*n* = 12). T0 (pre‐exercise) from V2 was used to compare resting pre‐ to post‐supplementation pulse wave analysis. Abbreviations: VO_2peak_, peak oxygen consumption; DEXA, dual‐energy x‐ray absorptiometry; T#, time point. Image created with a licensed version of BioRender.

This study is a sub‐analysis of a parallel‐arm randomized, double‐blind, placebo‐controlled trial (NCT05221905). Participant visits are outlined in Figure 1. Visits consisted of an initial screening visit, with measures of participant characteristics, resting blood samples and a maximal cycling exercise test to determine the lactate threshold and peak oxygen consumption (${\dot{V}}_{O_{2}\mathrm{peak}}$) (used to define subsequent exercise intensities and caloric expenditures in subsequent testing visits).

Following the screening visit, participants were randomized into one of two parallel treatment allocations that were maintained throughout subsequent testing visits: (1) 13 mmol nitrate in the form of 140 mL of beetroot juice (BRJ); or (2) an identical placebo (PL) with the nitrate extracted (<0.1 mmol nitrate) (see section 2.4). Each participant completed three experimental (testing) visits in a random order: (1) a non‐exercise control (CON); (2) moderate‐intensity exercise (MIE); and (3) high‐intensity exercise (HIE) (Figure 1). All testing visits were conducted ≥48 h apart but within a 2 week period.

Before all physical and physiological testing, participants abstained from all food or drinks other than water for ≥6 h, caffeine for ≥12 h, and exercise or alcohol for ≥24 h. Participants were encouraged to maintain their physical activity and dietary habits throughout the study period, except for the avoidance of high‐nitrate foods during supplementation. All procedures were approved by the Institutional Review Board at the University of Virginia (HSR#210326), and the study was conducted in accordance with the *Declaration of Helsinki*. All participants provided written informed consent.

### 2.2 Participants

Twenty‐four (12 per treatment arm) oestrogen‐deficient PMFs were recruited from the University of Virginia and surrounding Charlottesville, VA area. Postmenopausal status was defined as the absence of menstruation for ≥12 months consecutively. Participants were sedentary or recreationally active (<3 days/week of exercise). Exclusion criteria included: (1) smoker; (2) use of hormone therapy in the last year; or (3) history of a hysterectomy or oophorectomy. Medications were allowed on a limited basis, provided they did not interfere with nitrate supplementation (i.e., nitrates, proton pump inhibitors or H_2_ blockers).

### 2.3 Screening (pre‐randomization) visits

Eligible participants identified following a telephone pre‐screening were invited to the Clinical Research Unit (CRU) at the University of Virginia School of Medicine, where they completed informed consent forms, had resting blood pressure, height and weight measured, and were evaluated by a study physician, who reviewed their time since menopause.

An intravenous catheter was placed in the antecubital fossa for serial blood lactate sampling during rest and exercise. An incremental symptom‐limited cycle ergometer exercise test was conducted at the Exercise Physiology Core Laboratory. Open‐circuit spirometry was used to measure respiratory gases (Viasys Vmax Encore, Yorba Linda, CA, USA). The exercise test involved repeated 3 min stages increasing by 20 W until participants reached volitional fatigue. All participants met the following predefined criteria for achieving ${\dot{V}}_{O_{2}\mathrm{peak}}$ specific for older females: (1) maximum heart rate (HR) within 10 beats/min of age‐predicted maximal HR; (2) respiratory exchange ratio > 1.10; and (3) a rating of perceived exertion > 17 (Edvardsen et al., 2014). Blood lactate was sampled in the last 30 s of each stage, analysed using a YSI 2900 D Biochemistry Analyzer (Yellow Springs, OH, USA), and the resultant data were plotted to determine the lactate threshold (LT) (Green et al., 1983).

On a separate day (≥48 h later), participants completed a dual X‐ray absorptiometry scan (Hologic Horizon) for the assessment of percentage body fat. Following this and after ≥10 min of supine rest in dark, temperature‐regulated conditions, aortic haemodynamics (pulse wave analysis) were measured by the same investigator (between 08.00 and 10.00 h, consistent within a participant) using a SphygmoCor Xcel model (AtCor Medical, Itaska, IL, USA) following the manufacturer's instructions. Briefly, central aortic pressure waveforms were derived from cuff pulsations recorded at the brachial artery (consistent arm within a participant). Analysis of the waveform provided parameters, including central systolic pressure, central pulse pressure, augmentation pressure and augmentation index. The Buckberg ratio was calculated by the software as the ratio of diastolic to systolic pressure–time integrals, providing an index of the balance between myocardial oxygen supply and demand. The diastolic pressure–time index (DPTI), representing oxygen supply, was obtained by calculating the area under the diastolic portion of the central aortic pressure waveform. The systolic pressure–time index (SPTI), representing oxygen demand, was obtained by calculating the area under the systolic portion of the waveform.

### 2.4 Supplementation protocol

After the screening visit, participants were randomized in a block‐matched fashion by age and ${\dot{V}}_{O_{2}\mathrm{peak}}$. Participants were assigned to either consume 13 mmol of nitrate daily in the form of nitrate‐rich BRJ (one 70 mL bottle morning and night) or a nitrate‐depleted beetroot juice (PL; <0.1 mmol) with identical taste and appearance. Both treatments were purchased from the same manufacturer (Beet It, James White Drinks Ltd, Ipswich, UK), and participants and study personnel were blinded to the allocation of the beverages. Participants were instructed to consume the beverages for 2 days prior to each testing visit and to drink two bottles 2 h before the start of each visit. This was to ensure optimal plasma nitrate and nitrite bioavailability (James et al., 2015; Wylie et al., 2013). Participants were provided with a form to log supplementation times and were instructed to return all bottles at each study visit. Participants were instructed to avoid high‐nitrate foods (i.e., spinach, arugula, celery, etc.) and grapefruit throughout the study period (O'Gallagher et al., 2021) and to avoid factors that could impact the oral and microbial environment, such as mouthwash or antibiotics (Bondonno et al., 2015; Kapil et al., 2013; Sundqvist et al., 2016).

### 2.5 Testing visits

Each of the three testing visits was identical except for the exercise intensity (HIE, MIE and CON). Participants arrived at the Department of Kinesiology after an overnight fast except for consuming 140 mL of BRJ or PL 2 h prior.

Participants rested in a supine position for ≥5 min, after which a resting blood sample was drawn into a nitrate‐free syringe (BD Leur‐Lok) and centrifuged immediately at 1500*g* for 5 min at 4°C. The plasma was then collected into nitrate‐free microtubes (Axygen Microtubes) and stored in −80°C freezers for later analysis of plasma nitrate (NO_3_^−^) and nitrite (NO_2_^−^). Plasma NO_3_^−^ and NO_2_^−^ samples were collected pre‐ and post‐supplementation whenever possible; however, a small number of pre‐supplementation samples were not obtained owing to occasional limitations in sample collection. As a result, sample sizes differ slightly between pre‐ and post‐supplementation time points. Post‐supplementation samples were collected successfully for all participants. NO analysis was performed by the same investigator. Plasma NO_3_^−^ and NO_2_^−^ were assessed via ozone‐based chemiluminescence using a Sievers NOA model 280i (GE Analytical Instruments, Boulder, CO, USA) as previously described (Pinder et al., 2008). Briefly, the NO_2_^−^ of the undiluted plasma samples was determined by its reduction to NO in the presence of glacial acetic acid and potassium iodide, as previously explained (Kenjale et al., 2011). Plasma samples for NO_3_^−^ analysis were deproteinized using cold ethanol precipitation in a 1:3 dilution (plasma:ethanol) followed by a 30 min incubation before being centrifuged at 14 000*g* for 10 min. The supernatant was removed for the subsequent NO_3_^−^ analysis in the presence of vanadium chloride in hydrochloric acid at 95°C.

Participants then underwent pulse wave analysis testing (labelled as time point 0 or T0) following the same guidelines as during the screening (pre‐screening) visit. Pulse wave analysis was repeated every 30 min following the experimental condition. T60 is the equivalent to 60 min after T0 and ∼25–33 min after completion of the MIE and HIE sessions, respectively. Subsequent 30 min time point tests are labelled T90, T120, T150 and T180 min.

Following pulse wave analysis, participants were unblinded to the exercise allocation for that specific day (HIE, MIE or CON). The CON session consisted of ∼30 min (to match the ∼30 min of exercise) of passive rest in a seated position. Exercise sessions consisted of calorically matched cycle ergometer (Lode Excalibur Sport, Groningen, The Netherlands) exercise at either MIE or HIE, with the goal of reaching 200 kcal of energy expenditure. Gas exchange and heart rate were recorded continuously during the exercise sessions (Cosmed Quark, Cosmed USA Inc., Concord, CA, USA). The time to expend 200 kcal was estimated based on the screening ${\dot{V}}_{O_{2}\mathrm{peak}}$ test, with real‐time adjustments made if energy during the exercise sessions was expended at a rate other than expected. MIE occurred at the power output associated with LT. HIE occurred at a power output associated with the power output that was 75% of the difference between LT and ${\dot{V}}_{O_{2}\mathrm{peak}}$ (75% Δ). If participants could not maintain this power output until 200 kcal was expended, power output was decreased to 50% Δ, then to 25% Δ if necessary.

### 2.6 Statistical analysis

All statistical analyses were performed in IBM SPSS (v.29.0.2.0), and figures were created in GraphPad Prism or a licensed version of BioRender. Statistical significance was set at *P* < 0.05 a priori. Data are presented as the mean ± SD unless otherwise noted.

Pre‐supplementation (screening visit) baseline characteristics were analysed by independent sample *t*‐tests to identify any differences between participants subsequently allocated to each treatment (Table 1).

**TABLE 1:** Baseline characteristics (from screening visit).

Variable	PL	BRJ	*P*‐value
*n*	12	12	
Age, years	61 ± 5	59 ± 5	0.418
Time since menopause, years	11 ± 6	9 ± 5	0.301
Systolic BP, mmHg	123 ± 12	127 ± 12	0.411
Diastolic BP, mmHg	75 ± 8	74 ± 7	0.585
Height, cm	162 ± 6	167 ± 7	0.056
Weight, kg	71 ± 14	78 ± 17	0.272
Body mass index, kg/m^2^	26.9 ± 5.4	28.0 ± 6.6	0.679
Body fat, %	36 ± 7	38 ± 6	0.426
${\dot{V}}_{O_{2}\mathrm{peak}}$, mL/kg/min	24 ± 4	23 ± 5	0.554
${\dot{V}}_{O_{2}}$ @ LT, mL/kg/min	15 ± 3	14 ± 3	0.792
Medications, (*n*)			
Levothyroxine	2	4	–
Lisinopril	0	1	–
Alendronate	1	0	–
Simvastatin	1	0	–
Abilify	1	0	–
β‐Blocker	1	1	–

*Note*: PL versus BRJ were compared with an independent samples *t*‐test and significance set at *P* < 0.05. n = 12 for all variables (except medications). Abbreviations: BP, blood pressure; BRJ, beetroot juice; LT, lactate; PL, placebo; ${\dot{V}}_{O_{2}}$, oxygen consumption.

**FIGURE 2:**
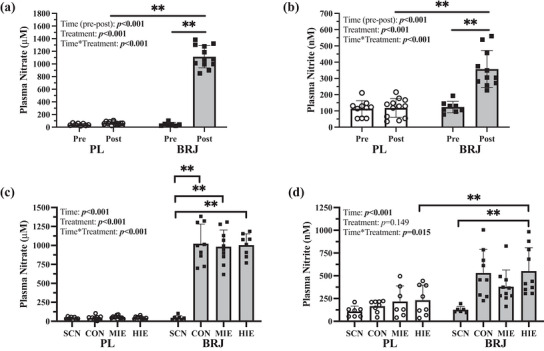
The effects of inorganic nitrate (BRJ) or placebo (PL) on plasma nitrate (NO_3_^−^) and plasma nitrite (NO_2_^−^). (a, b) Concentrations of plasma NO_3_^−^ (a) and NO_2_^−^ (b) are shown after 3 days of supplementation. (c, d) Concentrations plasma NO_3_^−^ (c) and NO_2_^−^ (d) measured pre‐exercise (T0) across the screening (SCN), control (CON), moderate‐intensity exercise (MIE) and high‐intensity exercise (HIE) visits. Data are presented as means ± SD. Analyses were performed using a two‐way repeated‐measures ANOVA with Bonferroni‐adjusted *post hoc* comparisons when appropriate. **P < 0.001 between groups. The time effect in (a, b) reflects pre–post changes within each treatment. For (c, d), time is defined as T0 at each testing visit. In each exercise condition, BRJ also resulted in higher plasma NO_3_^−^ concentrations compared with PL (all *P* < 0.001; data not shown for clarity). Sample sizes were as follows: (a, b) nitrate, Pre‐PL = 8, Post‐PL = 12, Pre‐BRJ = 7, Post‐BRJ = 12; nitrite, Pre‐PL = 10, Post‐PL = 12, Pre‐BRJ = 8, Post‐BRJ = 11; (c, d) nitrate, PL SCN = 8, CON = 10, MIE = 11, HIE = 11; BRJ SCN = 7, CON = 9, MIE = 10, HIE = 8; nitrite, PL SCN = 7, CON = 8, MIE = 7, HIE = 8; BRJ SCN = 6, CON = 9, MIE = 10, HIE = 10.

Post‐supplementation (BRJ or PL) characteristics were assessed at rest (T0) at the first testing visit, regardless of and prior to the first randomized exercise bout (CON, MIE or HIE). These resting post‐supplementation values were compared with the pre‐supplementation resting values obtained during the screening visit. Specifically, a two‐way repeated‐measures ANOVA was used to examine differences in plasma NO_3_^−^ and NO_2_^−^ concentrations (Figure 2a,b), and resting aortic haemodynamics and stiffness [systolic and diastolic blood pressure, pulse pressure, mean arterial pressure, heart rate, augmentation pressure, augmentation index adjusted to 75 beats/min (aAIx75), Buckberg ratio and reflection magnitude; Table 2]. To ensure that there were no differences between each of the three testing visits, we compared T0 resting values from each visit (CON vs. MIE vs. HIE) using a two‐way repeated‐measures ANOVA for plasma N‐oxides (Figure 2c,d) and aortic haemodynamics (Table 3). Owing to the randomized repeated‐measures design and incomplete datasets in a small number of participants, sample sizes differed slightly between analyses including all time points and those restricted to pre‐ versus post‐supplementation comparisons.

**TABLE 2:** Comparison of resting pre‐supplementation (screening) and testing visit post‐supplementation (PL vs. BRJ) resting (T0) aortic haemodynamics and stiffness.

	PL		BRJ		Time effect	Treatment effect	Interaction
Variable	Pre‐PL (screen)	Post‐PL (T0)	Pre‐BRJ (screen)	Post‐BRJ (T0)			
SBP, mmHg	113 ± 9	111 ± 8	120 ± 12	112 ± 13	0.078	0.382	0.275
DBP, mmHg	77 ± 7	76 ± 5	76 ± 7	75 ± 7	0.828	0.525	0.642
Pulse pressure, mmHg	35 ± 5	35 ± 5	44 ± 10#	37 ± 10*	**0.008**	0.129	**0.020**
MAP, mmHg	91 ± 8	89 ± 6	91 ± 8	88 ± 8	0.303	0.870	0.862
Heart rate, beats/min	61 ± 7	61 ± 7	56 ± 7	57 ± 9	0.678	0.162	0.910
Augmentation pressure, mmHg	11 ± 6	12 ± 5	13 ± 6	10 ± 4	0.558	0.950	0.089
Augmentation index @ HR 75 beats/min, %	22 ± 15	27 ± 14	19 ± 10	17 ± 7	0.357	0.208	0.170
Buckberg subendocardial viability ratio, %	145 ± 23	157 ± 22^*^	150 ± 17	158 ± 23	**0.005**	0.625	0.811
Reflection magnitude, %	64 ± 10	65 ± 10	65 ± 10	66 ± 9	0.371	0.888	0.928

*Note*: Results of a two‐way repeated‐measures ANOVA examining the effects of BRJ supplementation (treatment) and measurement period (time pre to post PL or BRJ supplementation) on resting aortic haemodynamics. Significance was set at *P* < 0.05, and significant effects are in bold. Pairwise comparisons were Bonferroni adjusted. *n* = 11 for all variables. Pre‐PL and pre‐BRJ values were obtained during the screening (pre‐supplementation) visit. Post‐PL and post‐BRJ were obtained at T0 (pre‐exercise) of testing visit 2.

Abbreviations: DBP, diastolic blood pressure; MAP, mean arterial blood pressure; SBP, systolic blood pressure.

*Significant difference compared with pre‐variable within treatment group.

#Significant difference compared with the pre‐variable in the opposite treatment group.

**TABLE 3:** Pre‐exercise (T0) aortic haemodynamics at baseline/pre‐supplemented and each post‐supplemented experimental visit.

Variable	Treatment	BL	CON	MIE	HIE	Time effect	Treatment effect	Interaction
aSBP, mmHg	PL	114 ± 9	110 ± 9	111 ± 7	112 ± 6	**0.030**	0.454	0.422
	BRJ	120 ± 11	113 ± 14^*^	115 ± 13	112 ± 110			
aDBP, mmHg	PL	79 ± 7	76 ± 7	77 ± 5	77 ± 8	0.098	0.320	0.776
	BRJ	75 ± 7	73 ± 6	76 ± 6	74 ± 7			
aPP, mmHg	PL	36 ± 6	35 ± 5	34 ± 5	35 ± 4	0.109	0.083	0.299
	BRJ	45 ± 10	40 ± 12	39 ± 9	38 ± 9			
aMAP, mmHg	PL	92 ± 7	89 ± 8	89 ± 5	90 ± 7	**0.046**	0.619	0.710
	BRJ	91 ± 8	87 ± 7	89 ± 9	87 ± 8			
aAIx75, %	PL	22 ± 15	26 ± 11	26 ± 13	29 ± 8	0.504	0.196	0.174
	BRJ	19 ± 11	21 ± 8	20 ± 11	19 ± 8			
Buckberg ratio, %	PL	147 ± 23	153 ± 24	160 ± 19	149 ± 15	0.137	0.869	0.446
	BRJ	148 ± 18	155 ± 24	155 ± 21	157 ± 24			
Reflection magnitude, %	PL	64 ± 10	68 ± 6	64 ± 11	66 ± 9	0.536	0.930	0.642
	BRJ	64 ± 11	66 ± 10	66 ± 12	67 ± 9			

*Note*: Repeated‐measures (time defined as each visit) two‐way ANOVAs were performed to compare the impact of supplementation (treatment effect) on aortic haemodynamic variables at baseline/pre‐supplementation and pre‐exercise of each following experimental visit. Significance was set at *P* < 0.05, and significant effects are in bold.

Abbreviations: aAIx75, aortic augmentation index adjusted for heart rate of 75 beats/min; aAP, aortic augmentation pressure; aDBP, aortic diastolic blood pressure; aMAP, aortic mean arterial pressure; aSBP, aortic systolic blood pressure; BL, baseline; BRJ, beetroot juice; CON, control session; HIE, high‐intensity exercise session; MIE, moderate‐intensity exercise session; Pb, reflected pulse pressure; Pf, forward pulse pressure; PL, placebo.

^*^Represents significance compared to BL. *n* = 10 for all groups.

A two‐way repeated‐measures ANOVA was used to examine whether exercise conditions (MIE vs. HIE) and treatment (BRJ vs. PL) were matched for caloric expenditure as planned. We also examined exercise oxygen consumption (${\dot{V}}_{O_{2}}$), heart rate, Borg rating of perceived exertion and exercise duration. When a significant main effect or interaction was detected, pairwise comparisons were performed using Bonferroni‐adjusted *post hoc* analysis (Table 4).

**TABLE 4:** Experimental exercise variables.

	Moderate intensity		High intensity		Intensity effect	Treatment effect	Interaction
Variable (aortic)	PL	BRJ	PL	BRJ			
Power, W (%Power_peak_)	53 ± 18 (39)	66 ± 22 (48)	113 ± 18* (84)	121 ± 32* (87)	**<0.001**	0.277	0.232
Average ${\dot{V}}_{O_{2}}$, mL/kg/min ($\%{\dot{V}}_{O_{2}\mathrm{peak}}$)	16 ± 4 (66)	16 ± 3 (68)	20 ± 4* (83)	20 ± 5* (85)	**<0.001**	0.668	0.882
HR, beats/min (% HR_peak_)	107 ± 22 (66)	115 ± 15 (71)	147 ± 10^*^ (90)	142 ± 8* (89)	**0.007**	0.849	0.904
Borg rating of perceived exertion	12 ± 1	12 ± 1	17 ± 1*	17 ± 1*	**<0.001**	0.879	0.942
Duration, min	38 ± 4	32 ± 8	29 ± 2*	26 ± 5*#	**<0.001**	**0.032**	0.353
Energy expenditure, kcal	198 ± 19	200 ± 13	200 ± 21	198 ± 16	0.951	0.953	0.165

*Note*: Results of a two‐way repeated‐measures ANOVA examining the effects of NO_3_^−^ supplementation (treatment) and exercise intensity (Intensity) on experimental exercise variables. Significance was set at *P* < 0.05 and significant effects are in bold. All sample sizes are *n* = 12 except the following: HR *n* = 9, duration BRJ *n* = 11, energy expenditure BRJ *n* = 11. Pairwise comparisons were Bonferroni adjusted.

*Significant difference within a treatment across exercise intensities.

#Significant difference between treatments within a given exercise intensity.

**FIGURE 3:**
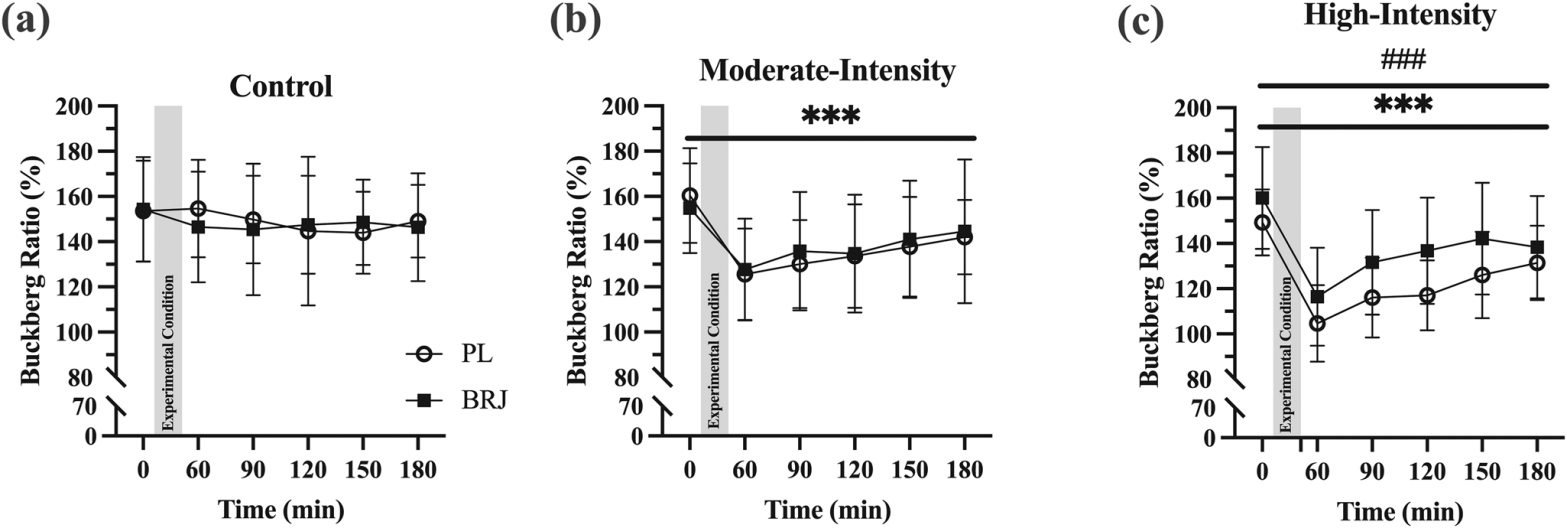
The effects of inorganic nitrate (BRJ) or placebo (PL) on Buckberg subendocardial viability ratio every 30 min following a resting control (a), moderate‐intensity exercise (b) and high‐intensity exercise (c) session. Data sets are means ± SD. Linear mixed‐effect models with repeated measures were performed. ***Significant (*p* < 0.001) time effect; ###significant (*P* < 0.001) treatment effect. Unless noted, all time points are *n* = 12: control group, PL = 11 (T120, T150 and T180 = 10), BRJ = 11 (T180 = 10); moderate intensity, PL = 12 (T150 and T180 = 11), BRJ = 12 (T180 = 11); high‐intensity, PL = 11 (T0‐T190 = 11), BRJ = 12 (T180 = 11). Abbreviations: BRJ, beetroot juice; PL, placebo.

The baseline (T0) Buckberg ratio was compared between treatment groups within each exercise intensity using independent samples *t*‐tests to assess for potential pre‐exercise differences. Postexercise Buckberg ratio was the main outcome of interest in this study and was examined by a linear mixed model to determine differences between time points (T0, T60, T90, T120, T150 and T180) and experimental conditions (fixed effects: exercise intensity and treatment combinations; Figure 3). Linear mixed models analysis was also performed for additional aortic haemodynamic variables (systolic and diastolic blood pressure, pulse pressure, mean arterial pressure, heart rate, augmentation pressure, augmentation index adjusted to 75 beats/min, and reflection magnitude) and reported in Tables 5, 6, 7.

**TABLE 5:** Aortic haemodynamics after resting control.

Control conditions		Time (min)						Time effect	Treatment effect	Interaction
Variable	Treatment	0	60	90	120	150	180			
aSBP, mmHg	PL	111 ± 8	115 ± 9	113 ± 10	117 ± 9	117 ± 9	118 ± 9	0.743	0.966	0.876
	BRJ	115 ± 14	116 ± 12	116 ± 10	116 ± 9	119 ± 13	110 ± 36			
aDBP, mmHg	PL	76 ± 7	77 ± 7	77 ± 7	79 ± 9	76 ± 7	80 ± 9	0.803	0.099	0881
	BRJ	73 ± 6	75 ± 5	77 ± 7	75 ± 7	77 ± 7	76 ± 6			
aPP, mmHg	PL	34 ± 5	37 ± 5	36 ± 5	38 ± 7	41 ± 4	39 ± 3	0.445	**0.006**	0.919
	BRJ	41 ± 12	41 ± 10	40 ± 7	41 ± 7	42 ± 9	44 ± 12			
aMAP, mmHg	PL	89 ± 8	90 ± 7	90 ± 8	92 ± 8	91 ± 8	94 ± 9	0.700	0.531	0.959
	BRJ	88 ± 8	90 ± 7	91 ± 8	90 ± 8	91 ± 9	91 ± 7			
aAP, mmHg	PL	12 ± 4	10 ± 3	10 ± 4	10 ± 4	11 ± 5	10 ± 4	0.457	0.350	0.963
	BRJ	12 ± 7	9 ± 5	8 ± 4	9 ± 5	9 ± 5	10 ± 7			
aAIx75, %	PL	27 ± 11	18 ± 10	19 ± 11	19 ± 22	17 ± 12	19 ± 13	**0.027**	0.120	0.973
	BRJ	20 ± 8	13 ± 11	11 ± 10	23 ± 38	11 ± 11	14 ± 10			
Reflection magnitude, %	PL	68 ± 6	65 ± 9	64 ± 7	65 ± 8	68 ± 8	64 ± 10	0.388	**0.041**	0.951
	BRJ	66 ± 9	61 ± 11	63 ± 9	61 ± 9	62 ± 10	60 ± 15			

*Note*: Linear mixed models with repeated effects (time) were performed to compare the impact of supplementation (treatment effect) on aortic haemodynamic variables during the resting control conditions. Significance was set at *P* < 0.05, and significant effects are in bold. Sample sizes: PL and BRJ T0–T120 *n* = 11, PL T120 *n* = 10, PL T150 *n* = 10, PL and BRJ T180 *n* = 10.

Abbreviations: aAIx75, aortic augmentation index adjusted for heart rate of 75 beats/min; aAP, aortic augmentation pressure; aDBP, aortic diastolic blood pressure; aMAP, aortic mean arterial pressure; aPP, aortic pulse pressure; aSBP, aortic systolic blood pressure; BRJ, beetroot juice; Pb, reflected pulse pressure; Pf, forward pulse pressure; PL, placebo.

**TABLE 6:** Aortic haemodynamics after moderate‐intensity exercise.

Moderate‐intensity exercise		Time (min)						Time effect	Treatment effect	Interaction
Variable	Treatment	0	60	90	120	150	180			
aSBP, mmHg	PL	109 ± 7	106 ± 7	106 ± 7	107 ± 8	108 ± 12	111 ± 9	0.186	0.110	0.981
	BRJ	114 ± 13	109 ± 10	106 ± 10	110 ± 11	110 ± 11	115 ± 15			
aDBP, mmHg	PL	76 ± 6	75 ± 6	73 ± 7	73 ± 7	75 ± 8	76 ± 8	0.493	0.579	0.996
	BRJ	76 ± 6	74 ± 6	72 ± 8	73 ± 7	73 ± 7	75 ± 7			
aPP, mmHg	PL	34 ± 5	31 ± 5	33 ± 5	34 ± 5	33 ± 5	36 ± 4	0.320	**0.006**	0.976
	BRJ	39 ± 9	35 ± 8	34 ± 7	37 ± 8	37 ± 8	39 ± 12			
aMAP, mmHg	PL	88 ± 6	88 ± 7	86 ± 7	86 ± 7	87 ± 10	88 ± 8	0.300	0.741	0.903
	BRJ	89 ± 8	88 ± 7	82 ± 9	86 ± 8	86 ± 7	89 ± 9			
aAP, mmHg	PL	10 ± 3	9 ± 5	8 ± 5	8 ± 5	7 ± 3	10 ± 4	**0.035**	0.224	0.931
	BRJ	11 ± 4	8 ± 6	6 ± 4	8 ± 4	7 ± 4	8 ± 7			
aAIx75, %	PL	25 ± 13	28 ± 13	20 ± 13	19 ± 13	16 ± 10	20 ± 11	**0.017**	**<0.001**	0.967
	BRJ	19 ± 10	18 ± 13	12 ± 10	13 ± 11	10 ± 11	10 ± 12			
Reflection magnitude, %	PL	65 ± 11	59 ± 13	58 ± 12	59 ± 9	57 ± 7	61 ± 11	0.170	0.485	0.889
	BRJ	65 ± 11	59 ± 7	56 ± 12	56 ± 12	60 ± 11	56 ± 16			

*Note*: Linear mixed model with repeated effects (time) were performed to compare the impact of supplementation (treatment effect) on aortic haemodynamic variables during the resting MIE conditions. No differences PL versus BRJ at T0 for each variable (independent *t*‐test). Significance was set at *P* < 0.05, and significant effects are in bold. Sample sizes: PL and BRJ T0–T120 *n* = 12, PL and BRJ T150–T180 *n* = 11.

Abbreviations: aAIx75, aortic augmentation index adjusted for heart rate of 75 beats/min; aAP, aortic augmentation pressure; aDBP, aortic diastolic blood pressure; aMAP, aortic mean arterial pressure; aPP, aortic pulse pressure; aSBP, aortic systolic blood pressure; BRJ, beetroot juice; Pb, reflected pulse pressure; Pf, forward pulse pressure; PL, placebo.

**TABLE 7:** Aortic haemodynamics after high‐intensity exercise.

High‐intensity exercise		Time (min)						Time effect	Treatment effect	Interaction
Variable	Treatment	0	60	90	120	150	180			
aSBP, mmHg	PL	112 ± 6	104 ± 8	98 ± 8	102 ± 7	107 ± 8	109 ± 8	**0.014**	**0.036**	0.834
	BRJ	112 ± 12	110 ± 16	106 ± 12	106 ± 13	110 ± 14	112 ± 12			
aDBP, mmHg	PL	77 ± 7	76 ± 5	68 ± 16	72 ± 7	74 ± 7	77 ± 7	0.272	0.944	0.662
	BRJ	75 ± 7	75 ± 9	73 ± 7	72 ± 6	75 ± 7	73 ± 7			
aPP, mmHg	PL	35 ± 4	28 ± 5	27 ± 5	30 ± 4	33 ± 2	32 ± 2	**0.041**	**0.002**	0.833
	BRJ	37 ± 10	35 ± 12	32 ± 9	34 ± 13	35 ± 12	39 ± 10			
aMAP, mmHg	PL	90 ± 7	89 ± 6	85 ± 4	85 ± 7	87 ± 7	90 ± 7	0.175	0.927	0.920
	BRJ	88 ± 8	90 ± 11	86 ± 9	85 ± 8	88 ± 8	88 ± 8			
aAP, mmHg	PL	13 ± 4	8 ± 7	7 ± 5	8 ± 5	7 ± 3	7 ± 4	**0.016**	0.905	0.858
	BRJ	11 ± 5	10 ± 7	7 ± 6	6 ± 6	8 ± 7	7 ± 6			
aAIx75, %	PL	31 ± 15	34 ± 16	26 ± 14	24 ± 15	19 ± 11	16 ± 14	**0.001**	**<0.001**	0.876
	BRJ	20 ± 8	26 ± 11	17 ± 15	12 ± 9	14 ± 10	11 ± 14			
Reflection magnitude, %	PL	66 ± 9	55 ± 8	57 ± 7	58 ± 8	60 ± 8	61 ± 6	**<0.001**	0.491	0.888
	BRJ	68 ± 9	50 ± 14	56 ± 10	57 ± 8	61 ± 13	58 ± 11			

*Note*: Linear mixed model with repeated effects (time) were performed to compare the impact of supplementation (treatment effect) on aortic haemodynamic variables during the resting HIE conditions. Significance was set at *P* < 0.05, and significant effects are in bold. Sample sizes: BRJ T0–T150 *n* = 12, BRJ T180 *n* = 11, PL T0–T180 *n* = 11.

Abbreviations: aAIx75, aortic augmentation index adjusted for heart rate of 75 beats/min; aAP, aortic augmentation pressure; aDBP, aortic diastolic blood pressure; aMAP, aortic mean arterial pressure; aPP, aortic pulse pressure; aSBP, aortic systolic blood pressure; BRJ, beetroot juice; Pb, reflected pulse pressure; Pf, forward pulse pressure; PL, placebo.

## 3 RESULTS

### 3.1 Participant characteristics and blood markers

Twenty‐four PMFs (12 per treatment arm) completed the study. Baseline characteristics, including age, time since menopause, blood pressure, body mass, body fat percentage and aerobic fitness, were similar between groups (Table 1). Plasma NO_3_^−^ (Pre, 45 ± 28 µM vs. Post, 1094 ± 153 µM, *P* < 0.001) and NO_2_^−^ (Pre, 130 ± 33 nM vs. Post, 366 ± 133 nM, *P* < 0.001) increased significantly following BRJ supplementation (Figure 2). In contrast, no changes were observed with PL for plasma NO_3_^−^ (Pre, 41 ± 16 µM vs. Post, 73 ± 19 µM, *P* = 0.745) or NO_2_^−^ (Pre, 114 ± 50 nM vs. Post, 125 ± 58 nM, *P* = 0.726).

### 3.2 Screening visit aortic variables

Resting central haemodynamics and stiffness variables taken at the screening visit (pre‐supplementation) and at T0 of the first testing visit (post‐supplementation with BRJ or PL) are presented in Table 2. Resting Buckberg ratio increased following 3 days of supplementation for both PL (*P* = 0.025) and BRJ (*P* = 0.051).

Additionally, at the screening visit (prior to randomization or treatment), resting aortic pulse pressure (aPP) was higher in participants later allocated to the BRJ group compared with those allocated to placebo (*P* = 0.029). After 3 days of supplementation (testing visit 1), in resting conditions (T0), aPP was significantly reduced from screening (pre‐supplementation) levels in the BRJ group only (*P* < 0.001). These two effects are likely to be related, because the interaction (*P* = 0.123) and time (*P* = 0.374) effects were no longer significant when screening (pre‐supplementation) aPP was included as a covariate in the analysis.

### 3.3 Exercise variables

Data from the experimental exercise bouts are presented in Table 3. As intended, energy expenditure did not differ between exercise conditions. Accordingly, power output (*P* < 0.001), steady‐state ${\dot{V}}_{O_{2}}$ (*P* < 0.001) and rating of perceived exertion (*P* < 0.001) were higher, whereas exercise duration was shorter (*P* < 0.001), during the HIE session. There was a treatment effect (*P* = 0.032) such that, at each exercise intensity, participants reached the targeted caloric expenditure more quickly following BRJ supplementation. However, this was not accompanied by a treatment effect on power output (*P* = 0.227) between PL and BRJ, indicating that total work was comparable across supplementation conditions.

### 3.4 Aortic haemodynamics across exercise conditions

Tables 5, 6, 7 present the aortic measures (systolic and diastolic blood pressure, pulse pressure, mean arterial pressure, heart rate, augmentation pressure, augmentation index adjusted to 75 beats/min and reflection magnitude) every 30 min after CON, MIE and HIE sessions, respectively.

After the resting control session (Table 5), aAIx75 declined over time (*P* = 0.027; time effect). Compared with PL, the BRJ condition showed lower reflection magnitude (*P* = 0.041) and higher aPP (*P* = 0.006; treatment effect). No interaction effects were observed.

After MIE (Table 6), both aAP (*P* = 0.035) and aAIx75 (*P* = 0.017) decreased over time (time effect). The BRJ condition was associated with lower aAIx75 (*P* < 0.001) and higher aPP (*P* = 0.006) compared with PL, without interaction effects.

Following HIE (Table 7), aSBP (*P* = 0.014), aPP (*P* = 0.041), aAP (*P* = 0.016), aAIx75 (*P* = 0.001) and reflection magnitude (*P* < 0.001) all declined over time (time effect). BRJ was associated with lower aAIx75 (*P* < 0.001), attributable, in part, to higher baseline values at T0. The aSBP (*P* = 0.036) and aPP (*P* = 0.002) were higher with BRJ compared with PL. No interaction effects were observed.

### 3.5 The Buckberg ratio

The effects of BRJ or PL on the Buckberg ratio at rest (T0) and following each of the three conditions (CON, MIE and HIE) are presented in Figure 3. Baseline (T0) subendocardial viability ratio did not differ between treatment groups within any exercise intensity (CON, *P* = 0.941; MIE, *P* = 0.517; HIE, *P* = 0.195). Although a numerically higher subendocardial viability ratio was observed in the BRJ group during HIE, this difference did not reach statistical significance. There were no differences over time or between treatments (BRJ vs. PL) for the CON condition. Both the MIE and HIE conditions showed a significant time effect (both *P* < 0.001) for both treatments, which was lowest at 60 min and increased back towards baseline over the next 2 h. *Post hoc* analyses revealed that, relative to T0, the Buckberg ratio was significantly reduced at T60 (both *P* < 0.001), T90 (MIE, *P* = 0.005; HIE, *P* < 0.001) and T120 (MIE, *P* = 0.012; HIE, *P* < 0.001), with reductions persisting at T150 (*P* = 0.025) and T180 (*P* = 0.022) in the HIE group.

In the PL arm of the trial, the Buckberg ratio was significantly reduced during HIE compared with both CON (*P* < 0.001) and MIE (*P* < 0.001). This difference between exercise conditions was not evident in the BRJ arm of the trial, although HIE remained lower than CON (*P* = 0.031). This effect of BRJ treatment was also observed within the HIE condition (*P* < 0.001), with the Buckberg ratio values being higher in the BRJ group compared with PL.

## 4 DISCUSSION

This randomized, double‐blind, placebo‐controlled study investigated the acute effects of exercise intensity and oral inorganic nitrate supplementation on the Buckberg ratio, a non‐invasive estimate of myocardial perfusion, in PMFs. The primary findings were as follows: (1) acute HIE resulted in a reduction in the Buckberg ratio (Figure 3c); and (2) inorganic NO_3_^−^ supplementation attenuated this postexercise decline following HIE (Figure 3c). Additionally, nitrate supplementation improved resting central haemodynamics, including reductions in central systolic blood pressure, augmentation pressure and pulse pressures (Table 2).

Overall, these findings build on our prior report that nitrate supplementation combined with HIE improved vascular function in this cohort (Hogwood et al., 2023), suggesting that it also enhances central haemodynamics by supporting myocardial supply–demand balance during cardiovascular stress in oestrogen‐deficient females. To our knowledge, this is the first study to examine the impact of nitrate supplementation and exercise intensity on the Buckberg ratio in PMFs.

### 4.1 Impaired myocardial perfusion and exercise Buckberg ratio response

The Buckberg ratio, the ratio of diastolic to systolic pressure–time integrals, is a non‐invasive estimate of myocardial perfusion, with lower values being associated with ischaemia and adverse cardiovascular outcomes (He et al., 2021; Pinheiro et al., 2025; Tsiachris et al., 2012). In our study, the Buckberg ratio declined following both MIE and HIE, with the largest reductions observed after HIE in the PL group (Figure 3). Although a minimal clinically important difference for the Buckberg ratio has not been established, reductions in this index reflect impaired myocardial oxygen supply–demand balance, and decreases of the order of ∼20%–25% have been reported in populations with compromised coronary perfusion (Xie et al., 2024). In this context, the magnitude of reduction (27%–30%) observed following HIE supports a physiologically meaningful perturbation in subendocardial perfusion. MIE did not significantly reduce the Buckberg ratio beyond the observed time effect, highlighting that exercise intensity, rather than duration, might be the primary driver of acute perfusion stress in PMFs. This intensity‐dependent response reflects the greater haemodynamic burden imposed at higher exercise intensities, when elevations in heart rate and systolic pressure shorten diastolic filling (the critical window for myocardial perfusion) and elevate myocardial workload (Doonan et al., 2011; Lundberg et al., 2008). PMFs might be particularly vulnerable in this setting owing to oestrogen deficiency, which impairs NO signalling, vasodilatory reserve and the ability to increase coronary blood flow (Carlini et al., 2024; Lundberg et al., 2008; Raj et al., 2023). BRJ supplementation attenuated the decline in the Buckberg ratio, such that the Buckberg ratio values following HIE were comparable to MIE. This suggests that exogenous NO buffered the adverse myocardial perfusion response to HIE. Mechanistically, this effect is consistent with the activation of the nitrate–nitrite–NO pathway, which is enhanced in conditions of reduced oxygen when endothelial nitric oxide activity is impaired (Lundberg et al., 2008), thereby potentially supporting coronary perfusion when demand is greatest. These findings indicate that nitrate supplementation might reduce acute perfusion vulnerability during vigorous activity, perhaps by enhancing coronary vasodilatation.

Although the baseline Buckberg ratio did not differ statistically between treatment groups, a numerically higher Buckberg ratio was observed in the BRJ condition during high‐intensity exercise. To ensure that this did not influence interpretation of the treatment effect, we performed a secondary analysis, expressing the Buckberg ratio as the change from baseline (ΔBuckberg ratio = baseline − time point) and evaluated responses using linear mixed models. In contrast to the analysis of absolute values, this approach did not reveal significant treatment or treatment × time effects, suggesting that the observed attenuation of the Buckberg ratio decline with BRJ might be sensitive to baseline variability in high‐intensity conditions. However, the Buckberg ratio is a physiologically meaningful index of myocardial oxygen supply–demand balance, and absolute values are commonly used to reflect this relationship. As such, the primary analysis focused on absolute responses, with the ΔBuckberg ratio analyses serving as a complementary sensitivity assessment.

### 4.2 Resting haemodynamic effects of inorganic nitrate supplementation

A strength of our design was the collection of resting central haemodynamic data in both an unsupplemented state and after 3 days of supplementation, enabling us to evaluate the vascular effects of inorganic nitrate supplementation independent of exercise. The Buckberg ratio increased from screening (pre‐supplementation) to T0 across both PL and BRJ groups, resulting in a significant time effect. However, there was no treatment effect or group × time interaction; the apparent change was probably driven by lower screening values in participants later randomized to PL, which were not statistically different from BRJ at baseline (Table 5). It is also possible that this observation reflects our study design, because participants consumed the assigned supplement shots ∼2 h prior to testing. Thus, they were not truly fasted at the post‐supplementation visits, and the carbohydrate content or juice matrix itself (rather than nitrate) might have contributed to the observed increases in the Buckberg ratio (Jovanovski et al., 2015).

**FIGURE A1:**
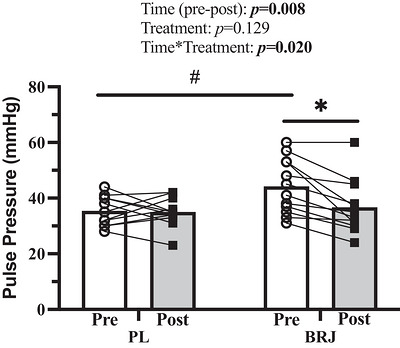
Aortic pulse pressure at rest pre‐ and post‐supplementation. Bars represent means, with individual data points connected. Shown are the results of a two‐way repeated‐measures ANOVA examining the effects of BRJ supplementation (treatment) and measurement period (time pre to post PL or BRJ supplementation) on resting pulse pressure. Significance was set at *P* < 0.05, and significant effects are in bold. Pairwise comparisons were Bonferroni adjusted. *n* = 11 for PL and *n* = 12 for BRJ. Pre‐PL and pre‐BRJ values were obtained during the screening (pre‐supplementation) visit. Post‐PL and post‐BRJ were obtained at T0 (pre‐exercise) of testing visit 2. *Significant difference compared with variable pre‐supplementation within treatment group. #Denotes a significant difference compared with the pre‐supplementation variable in the opposite treatment group. Abbreviations: BRJ, beetroot juice; PL, placebo.

At rest, pre‐supplementation, aortic pulse pressure was higher in the BRJ group (*P* = 0.029). Given that participants were matched for key baseline characteristics (e.g., age, endothelial function and fitness), and no associations were observed between resting pulse pressure and indices of arterial stiffness or participant characteristics, this difference is likely to reflect random variation introduced by group allocation in a modest sample size rather than a true physiological difference (Figure A1). Following supplementation, aortic pulse pressure was significantly reduced, consistent with prior trials demonstrating that dietary nitrate preferentially lowers central pressures in individuals with elevated vascular load (Mattos et al., 2023; Pinheiro et al., 2025). However, because baseline aortic pulse pressure was higher in the BRJ group at screening, these participants might have had greater capacity for decline. Taken together, these findings suggest that inorganic nitrate acutely improves aortic compliance and reduces central pressure pulsatility in oestrogen‐deficient PMFs, probably through enhanced NO‐mediated vasodilatation and reduced wave reflection. Recent randomized controlled trials in older adults and PMFs support these effects, showing improved aortic compliance, reduced wave reflection and enhanced myocardial perfusion efficiency at rest following nitrate supplementation (He et al., 2021; Hogwood et al., 2023; Kim et al., 2019). Such haemodynamic improvements are physiologically relevant for the Buckberg ratio, because lower aortic pulse pressure prolongs the diastolic perfusion window.

### 4.3 Limitations and future directions

Although our study provides new insight into the interplay between exercise intensity, nitrate supplementation and the Buckberg ratio in PMFs, several limitations should be noted. The sample size was modest, and although the randomized, repeated‐measures design improved internal validity, larger trials are warranted to confirm these findings. Furthermore, although the Buckberg ratio is a validated estimate of myocardial perfusion (Xie et al., 2024), future studies incorporating direct imaging techniques might further elucidate the clinical implications of these responses.

Long‐term studies are needed to determine whether repeated bouts of exercise (e.g., exercise training) combined with nitrate supplementation lead to sustained improvements in myocardial perfusion and cardiovascular outcomes in PMFs. Additionally, the dose–response relationship of nitrate relative to body size, in addition to interindividual variability in nitrate reduction capacity, warrants further exploration.

**FIGURE A2:**
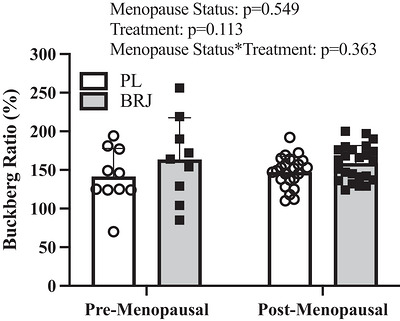
The Buckberg ratio at rest across menopausal status and treatment conditions. Values represent estimated marginal means ± SD. There were no significant main effects of menopausal status or treatment, and no interaction between factors. Sample sizes: premenopausal *n* = 10; postmenopausal *n* = 24. Abbreviations: BRJ, beetroot juice; PL, placebo.

The present study did not include a premenopausal group for comparison. However, exploratory analyses incorporating a premenopausal cohort suggested no significant differences in resting Buckberg ratio or treatment response between groups (Figure A2); however, these findings should be interpreted cautiously, given differences in study design and the absence of an exercise stimulus in the premenopausal cohort. We recognize the lack of a directly comparable, prospectively collected premenopausal group within the same experimental framework as a limitation, which precludes our ability to disentangle fully the independent effects of age and menopausal status on the Buckberg ratio responses.

An additional consideration is the timing of nitrate supplementation and blood sampling. Beetroot juice was administered ∼2 h prior to exercise to align with the known peak in plasma NO_3_^−^ and NO_2_^−^ concentrations, thereby maximizing NO bioavailability during the exercise bout. Although plasma NO_3_^−^ and NO_2_^−^ concentrations were confirmed prior to exercise and previously shown to remain elevated immediately following exercise in this cohort (Hogwood et al., 2023), blood samples were not collected at each postexercise pulse wave analysis time point. Therefore, we cannot definitively determine the temporal relationship between circulating NO metabolites and the recovery of postexercise haemodynamics across the full 3 h assessment period.

## 5 CONCLUSION

In summary, acute exercise reduces the Buckberg ratio in PMFs in an intensity‐dependent manner, with HIE producing the most pronounced decline. Inorganic nitrate supplementation attenuates this response, particularly following HIE, and might improve resting aortic haemodynamics. These findings highlight the potential of inorganic nitrate as a non‐pharmacological strategy to maintain myocardial oxygen supply–demand balance during physiological stress in PMFs, a population vulnerable to coronary heart disease.

## AUTHOR CONTRIBUTIONS

Cassandra C. Derella conceived the primary hypothesis, interpreted the results, prepared the figures and drafted the manuscript. Austin C. Hogwood, Joaquin Ortiz de Zevallos, Kaitlin M. Love, Arthur Weltman and Jason D. Allen all worked to develop the original research project (of which this is a sub‐analysis) and design. Austin C. Hogwood, Joaquin Ortiz de Zevallos and Nathan R. Weeldreyer performed experiments and collected the data. All authors edited, revised and approved the final version of the manuscript and agree to be accountable for all aspects of the work in ensuring that questions related to the accuracy or integrity of any part of the work are appropriately investigated and resolved. All persons designated as authors qualify for authorship, and all those who qualify for authorship are listed.

## CONFLICT OF INTEREST

None declared.

## GENERATIVE AI STATEMENT

AI was not used in the development of this project.

## Data Availability

Data are available upon request.

## Exploratory comparison by menopausal status

To address whether the observed responses in the Buckberg ratio might differ by menopausal status (Figure A2), an exploratory analysis was conducted, incorporating a cohort of premenopausal females from a separate study (IRB #21983) within our laboratory. Given differences in study design, specifically, a randomized crossover design in the premenopausal cohort and a parallel‐group design in the postmenopausal cohort, a linear mixed‐effects model was used, with subject included as a random effect to account for repeated‐measures within the premenopausal group while retaining all available observations.

At rest, there was no significant main effect of menopausal status on the Buckberg ratio (*P* = 0.549). There was also no significant main effect of treatment (BRJ vs. PL, *P* = 0.113), although a non‐significant trend towards an increased Buckberg ratio with BRJ was observed. Importantly, there was no significant interaction between menopausal status and treatment (*P* = 0.363), indicating that the effect of BRJ on resting Buckberg ratio did not differ between pre‐ and postmenopausal females.

These findings suggest that, in resting conditions, the vascular response to BRJ might not be modified strongly by menopausal status. However, these analyses should be interpreted with caution, given the exploratory nature of cross‐cohort comparisons, differences in study design (crossover vs. parallel), and the absence of an exercise stimulus in the premenopausal cohort.
